# Comment: ineffectiveness of hemoadsorption in large animals with abdominal sepsis—a randomized controlled porcine study

**DOI:** 10.1186/s40635-025-00764-6

**Published:** 2025-07-30

**Authors:** Gerd Klinkmann, Matteo Marcello, Faeq Husain-Syed, Gonzalo Ramírez-Guerrero, Thiago Reis, Claudio Ronco

**Affiliations:** 1 Department of Anaesthesiology, Intensive Care Medicine and Pain Therapy, University Medical Center Rostock, Rostock, Germany; 2 Department of Extracorporeal Immunomodulation, Fraunhofer Institute for Cell Therapy and Immunology, Rostock, Germany; 3 International Renal Research Institute of Vicenza (IRRIV), Vicenza, Italy; 4 Department of Nephrology, Dialysis and Kidney Transplantation, San Bortolo Hospital, Vicenza, Italy; 5 Pulmonology and Critical Care Medicine, Member of the German Centre for Lung Research, University Hospital Giessen and Marburg, Giessen, Germany; 6 Nephrology Service, Las Higueras Hospital, Talcahuano, Chile; 7 Division of Nephrology, University of São Paulo Medical School, São Paulo, Brazil

We read with interest the recent study by Tegl et al. on hemoadsorption using a styrene–divinyl benzene resin in a porcine sepsis model [1]. While the work contributes to the debate on cytokine removal in sepsis, key methodological issues deserve clarification.

First, the adsorber used has minimal endotoxin affinity, yet the Gram-negative setting may wrongly imply endotoxin clearance—underscoring the need to clarify adsorption specificity.

Second, the authors exposed healthy controls to hemoadsorption [1]. Clinical practice never calls for extracorporeal cytokine removal in non-inflamed individuals, and the intervention itself provoked vasoplegia, cytopenias, and a cytokine surge—effects likely reflecting circuit–blood interactions rather than device-specific toxicity. In contrast, maintenance dialysis patients receiving hemoadsorption show no such effects [2], suggesting these findings reflect artificial, non-clinical conditions.

Third, hemoadsorption began while IL-6 and other cytokines were still low, with IL-6 rising even in sham animals. Since clearance is concentration-dependent, early use limits efficacy. IL-6’s − 15 h half-life and complex kinetics make 24 h measurements unreliable [3]. Human data show cytokine levels drop 50–70% when hemoadsorption starts during high inflammation, without hemodynamic issues [4]. This study may thus reflect sub-optimal conditions.

Fourth, the study lacked a priori power analysis. ARRIVE 2.0 guidelines highlight this as essential to ensure reliable results, especially in variable models like sepsis [5]. This omission limits the certainty of interpreting negative results, especially considering the complexity and variability inherent in sepsis models.

Fifth, the absence of antibiotics and source control limits clinical relevance. The current guidelines and modeling standards (e.g., MQTiPSS) recommend replicating standard care to enhance translational value [6, 7]. While logistically simpler, omitting these measures likely amplified cytokine responses beyond typical clinical scenarios.

Sixth, fluid resuscitation was excessive. Infusions and boluses resulted in net balances up to 9.4 mL/kg/h—equating to 16–19 L over 36 h—far exceeding goal-directed protocols. Such overload is associated with edema, organ dysfunction, and higher mortality, potentially obscuring any hemodynamic benefit of hemoadsorption [8].

Seventh, adsorber use did not reflect current best practices, as a single cartridge was maintained for 24 h. Consensus guidelines recommend exchanges every 6–12 h to prevent saturation and mediator rebound [9]. This likely limited efficacy. The ADQI 30 consensus also emphasizes saturation as a critical factor in hemoadsorption study design and application [10]. Future studies should follow these recommendations to ensure accurate biological assessment.

Finally, the relatively brief follow-up of 24 h limits assessment of delayed physiological responses critical in evaluating sepsis interventions. Literature reviews indicate that 36–48 h or even longer observation periods better capture organ dysfunction trajectories and delayed treatment effects in large animal sepsis models [8, 11]. Extending follow-up would provide deeper insights into the medium-term benefits or potential harms associated with hemoadsorption.

In summary, Tegl et al. offer meaningful contributions to the field of hemoadsorption in sepsis. Future studies will benefit from integrating standard care, sound statistical planning, optimized device protocols, and extended follow-up. We commend the authors and hope these reflections help refine future experimental approaches.

## Data Availability

The datasets used and analyzed during the current study are available from the corresponding author on reasonable request.
